# CD133-positive dermal papilla cells are a major driver in promoting hair follicle formation

**DOI:** 10.21203/rs.3.rs-5054470/v1

**Published:** 2025-04-23

**Authors:** Huangying Zhao, Linli Zhou, Lindsey Siegfried, Dorothy Supp, Steven Boyce, Thomas Andl, Yuhang Zhang

**Affiliations:** University of Cincinnati College of Pharmacy; University of Cincinnati College of Pharmacy; University of Cincinnati College of Pharmacy; University of Cincinnati Medical Center; University of Cincinnati Medical Center; University of Central Florida Burnett School of Biological Sciences; University of Cincinnati College of Pharmacy

**Keywords:** hair follicle, tissue engineering, keratinocytes, fibroblasts

## Abstract

A major contributing factor to the failure of cell-based human hair follicle (HF) engineering is our inability to cultivate highly specialized, inductive mesenchymal fibroblasts, which reside in a unique niche at the HF base, called the dermal papilla (DP). We and other groups have discovered a unique DP fibroblast subpopulation that can be identified by the cell surface marker CD133. However, the biological difference between CD133-positive (CD133+) and CD133-negative (CD133−) DP cells remains unknown. Using a newly developed double fluorescent transgenic mouse strain, we isolated CD133 + and CD133− DP cells from mouse anagen HFs. In monolayer culture, both DP populations gradually lost expression of the anagen DP signature gene, versican. When maintained in three-dimensional spheroid culture, versican expression was restored in both CD133 + and CD133− DP cells. Importantly, CD133 + DP spheroids appeared more compact, showed stronger alkaline phosphatase staining (AP), and expressed higher levels of DP signature genes. In *in vivo* skin reconstitution assays, mice grafted with CD133 + DP spheroids grew more hairs in healed wounds than those grafted with CD133− DP spheroids. The data underscore the importance of CD133 + DP cells as a driver of HF formation, which may present a unique opportunity to improve the use of human DP cells in tissue-engineered skin substitutes (TESS).

## Introduction

The development of tissue-engineered skin substitutes (TESS) has provided significant medical benefits for patients with extensive, deep skin loss^1,2^. Nevertheless, their application has been unsatisfactory due, in part, to compromised physiological functions resulting from missing hair follicles (HFs) and other skin appendages^3^. In addition, hair loss disorders, such as androgenic alopecia and early graying, can be psychologically devastating to affected individuals. Undoubtedly, understanding how to induce HF formation and promote hair growth in TESS could likely benefit patients with life-threatening wounds or disfiguring hair loss. Although epithelial keratinocytes are known to be the primary builder that generates HF structures and fibers, the key to controlling skin repair and appendage formation is epithelial-mesenchymal interactions (EMIs) between keratinocytes in the epithelial compartment and their immediate dermal microenvironment^4^. The major cell type that constitutes a unique dermal “niche” is fibroblasts of mesenchymal origin, which mainly reside in the dermal papilla (DP)^5^. After HF morphogenesis, DP cells continue to provide inductive signaling to drive the proliferation and differentiation of surrounding hair matrix keratinocytes, forming multiple layers of the outgrowing hair shaft and the inner root sheath^6^. Thus, the DP is an essential component in our efforts to generate fully functional TESS for burn victims as well as to treat alopecia. However, the clinical application of human DP fibroblasts to induce HF formation has been largely unsuccessful. First, it has been repeatedly demonstrated that fibroblasts isolated from microdissected human DPs quickly lose hair inductivity during *ex vivo* expansion and fail to produce HFs^6^. Second, DP fibroblasts proliferate slowly in *ex vivo* culture, failing to generate sufficient numbers of cells needed for HF engineering^7^. There is, therefore, a critical need and potentially a high reward in identifying novel, effective approaches to generating inductive DP fibroblasts for HF generation.

Although the DP appears to be a relatively static population of cells, the number of cells in the DP increases from telogen to anagen^8,9^. Furthermore, a recent report suggested that the DP cell number specifies the size and shape of murine pelage hairs and their cycling properties^10^. Several markers specifically demarcate the DP niche in the skin. However, only a few of these markers have proven to be useful for studying DP cells. CD133 has been identified as a surface marker in a subpopulation of DP cells during the anagen phase of murine HFs^11^. Further studies showed that CD133 + DP cells isolated from embryonic or adult DPs have the ability to induce new HFs *in vivo*^11,12^. We previously reported that the activation of canonical Wnt/b-catenin signaling in CD133 + DP cells accelerates postnatal hair growth and increases HF induction^13,14^. Based on these findings, we hypothesize that the heterogeneous DP niche consists of active (inductive) DP cells, which are CD133+, and static (responsive) DP cells, which are CD133−. Nevertheless, it remains to be determined whether CD133 indeed marks a major inductive DP subpopulation that functions to stimulate responsive DP cells and keratinocytes for HF induction.

A common issue for all previously published studies is that CD133− cells used for comparison are not definitively CD133− DP fibroblasts due to the lack of genetic tools that specifically target DP cells. Thus, DP heterogeneity cannot be properly dissected and assessed. To address this limitation, we established a triple transgenic mouse strain that allows us to isolate CD133 + and CD133− DP cells from mouse HFs for evaluation by specifically labeling DP fibroblasts with enhanced green fluorescent protein (EGFP). A detailed characterization confirmed DP heterogeneity and showed that CD133 + and CD133− DP cells indeed possess different characteristics. More importantly, CD133 + DP cells harbor unique biological properties in driving HF formation and may be useful for maintaining human DP inductivity, thus benefiting human HF engineering.

## Results

### Tracing and isolation of CD133 + and CD133− DP cells

**Figure 1:**
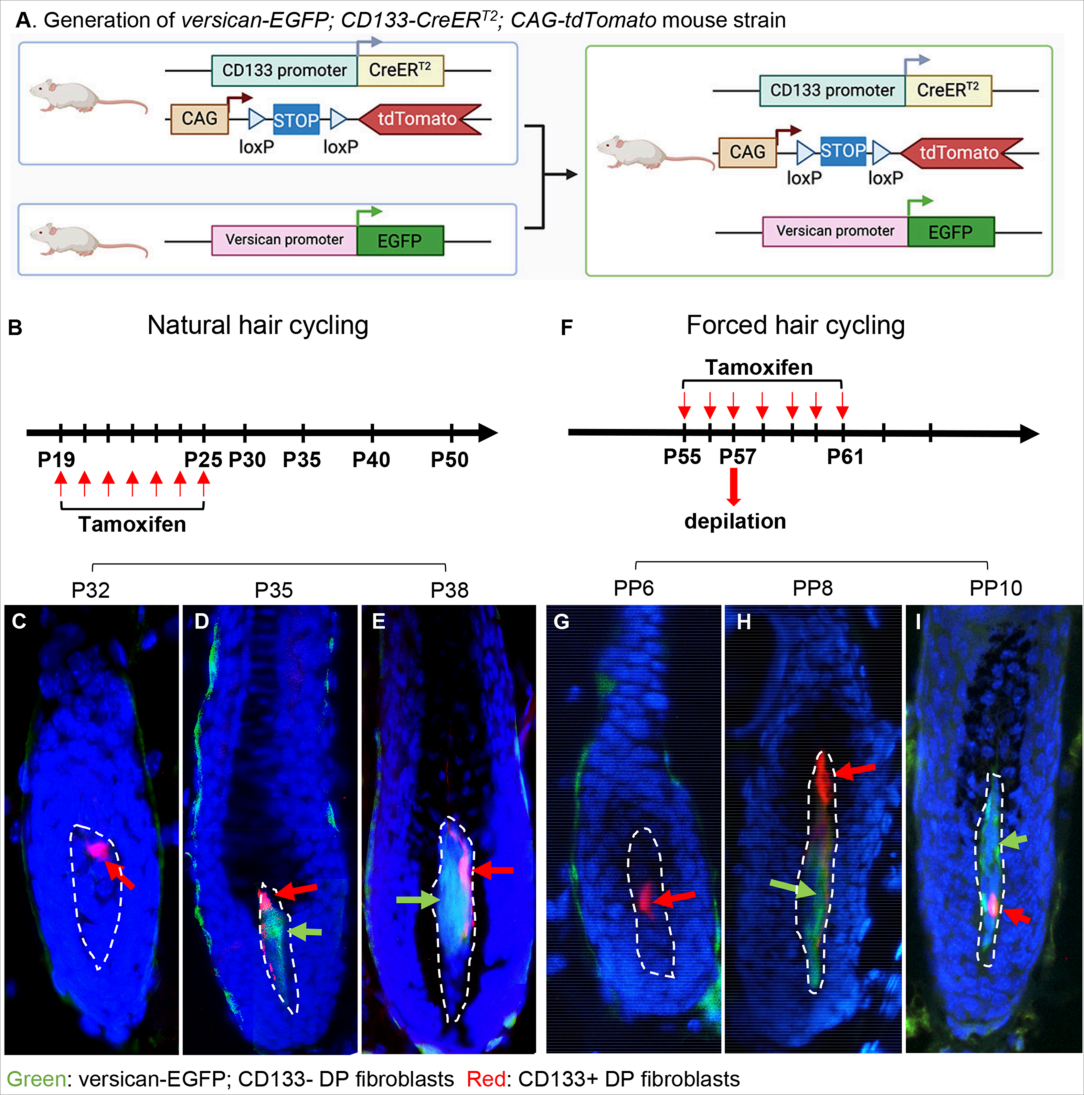
Heterogeneous DP populations marked by versican and CD133 expression in postnatal murine hair follicles. **A.** Generation of the *versican-EGFP*; *CD133-CreER*^*T2*^; *CAG-tdTomato* triple transgenic mouse strain. The illustration shows the process of crossing *versican-EGFP* transgenic mice with *CD133-CreER*^*T2*^; *CAG-tdTomato* transgenic mice and was created on BioRender.com. **B**. The time scheme of tamoxifen administration for inducing tdTomato expression during the natural hair cycle. Red arrows indicate the days when tamoxifen was administrated to recipient mice starting P19. **C-E.** Frozen sections of back skin biopsies at P32, P35, and P38 during the natural hair cycle showing the labeling of DP fibroblasts in each HF by green EGFP expression driven by the versican promoter and red tdTomato expression driven by CD133-driven Cre-mediated recombination. At each time point, at least four mice were analyzed. **F.** The time scheme of tamoxifen administration for inducing tdTomato expression during the forced hair cycle by hair plucking. Red arrows indicate the days when tamoxifen was administrated to recipient mice starting P55. **G-I.** Frozen sections of skin biopsies collected from the hair-plucking area at postpluck days 6,8, and 10 showing the labeling of DP fibroblasts in each HF by green EGFP expression driven by the versican promoter and red tdTomato expression driven by CD133-driven Cre-mediated recombination. At each time point, at least three mice were analyzed. CD133+ DP cells are red fluorescent cells and indicated by red arrows, and versican-EGFP+; CD133− DP cells are green fluorescent cells and indicated by green arrows.

The DP niche is known to be heterogeneous and exhibits hair cycle-associated plasticity^6^. During the anagen phase of fully developed HFs, CD133 expression only appears in a subpopulation of DP cells, and a large portion of cells in the DP remain CD133−^11^. To understand the characteristic differences between CD133 + and CD133− DP cells and to develop a feasible approach to isolate CD133− DP cells for comparison, we established a triple *versican-GFP; CD133-CreER*^*T2*^; *CAG-tdTomato* transgenic mouse strain by crossing versican-EGFP transgenic mice with *CD133-CreER*^*T2*^; *CAG-tdTomato* transgenic mice (Fig. 1A). In *versican-EGFP* transgenic mice, DP fibroblasts in anagen are labeled with EGFP under the control of a versican promoter, which is specifically expressed in the DP during anagen^15,16^. The unique CD133 + DP fibroblast population is tagged with a second fluorescent marker, the red fluorescent protein tdTomato, which is inducibly expressed via Cre-mediated recombination following tamoxifen treatment.

To better understand the dynamics between versican and CD133 expression in DP fibroblasts, the activities of CD133 and versican were analyzed in both natural (Fig. 1B) and forced hair cycles (Fig. 1F). Interestingly, the expression of CD133 indicated by red fluorescence was always seen first (Fig. 1C, G). As anagen progressed, versican expression gradually increased in the DP. As shown in Fig. 1D–E, during the natural hair growth stages between postnatal day 35 (P35) and P40, there was broad expression of green fluorescence driven by the versican promoter in DP fibroblasts; however, red fluorescence could only be detected in a small population of DP cells. We also examined their expression in plucking-induced hair growth. Similarly, red fluorescence-tagged CD133 + DP cells began to appear in the DP during early anagen on postpluck day 6 (PP6) before versican+ (GFP+) cells were observed in the DP (Fig. 1G). The population of CD133 + DP cells expanded until the full anagen stage when versican was widely expressed (Fig. 1H–I). By the time, two populations of DP fibroblasts, versican+; CD133 + DP cells (green+; red+) and versican+; CD133− (green+; red−) DP cells, were observed in the DP and could be readily isolated based on fluorescence. However, it appears that red fluorescent CD133 + DP cells were not maintained through the hair follicle telogen phase and need to be regenerated during each hair cycle (Fig. S1A-E). To validate green+; red + DP cells are indeed CD133 + and green+; red− DP cells are CD133−, we stained the PP8 hair follicle tissue sections using an allophycocyanin (APC)-labeled anti-CD133 antibody. As shown in Fig. S2, only red fluorescent cells in the DP region (C, G, K) overlapped with cells that were positive for APC staining (D, H, L). To simplify the terminology, two DP cell populations will be referred to as CD133 + and CD133− DP cells hereafter.

**Figure 2:**
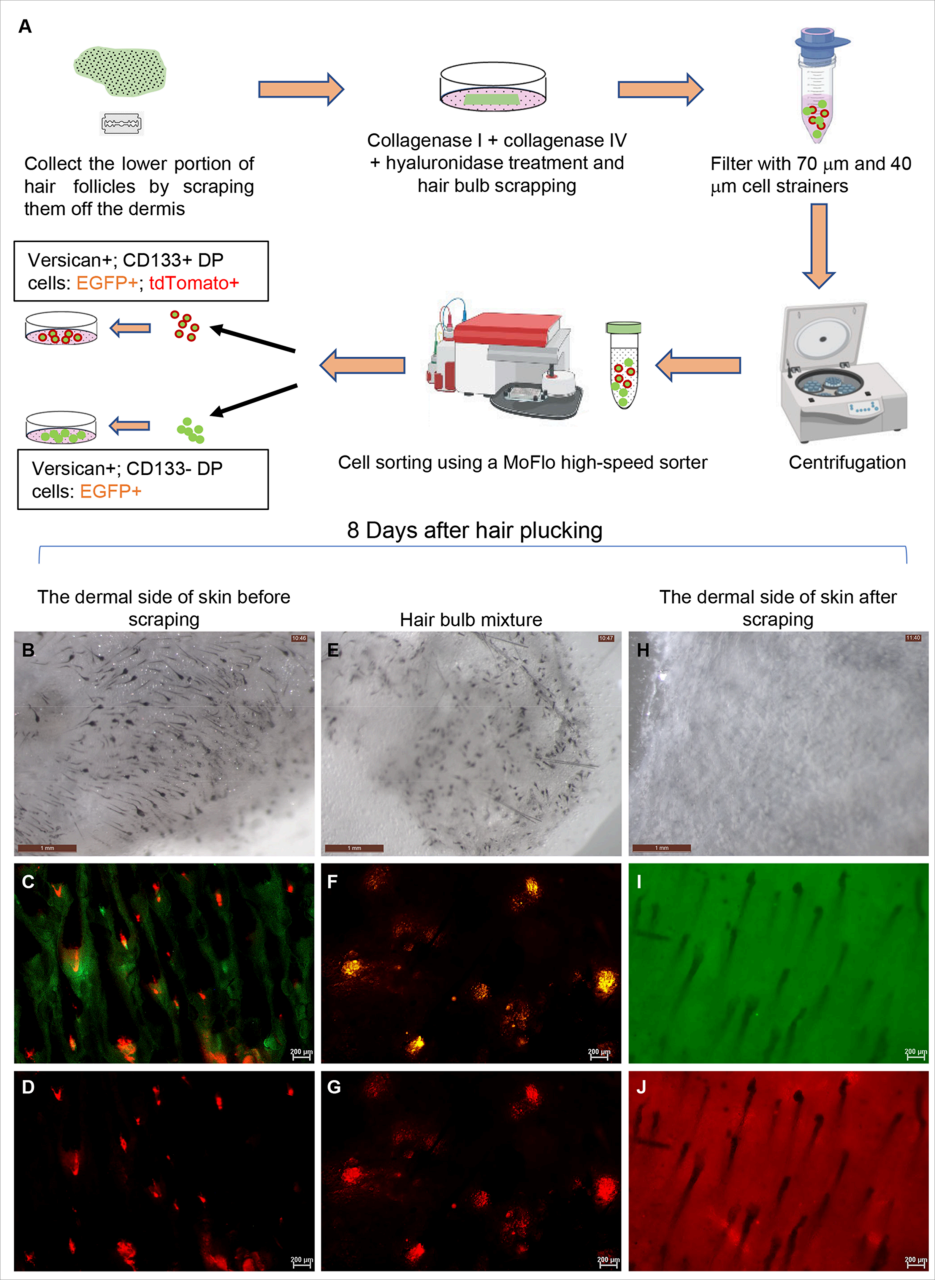
Isolation of versican+; CD133+ and versican+; CD133− DP cells from anagen hair follicles. **A.** Process of isolating versican+; CD133+ and versican+; CD133− DP cells from mouse HFs based on green and red fluorescence using FACS. **B-D**. Hair bulbs can be easily seen on the dermal side of the collected skin biopsy eight days after hair plucking. Red and green fluorescence were visible in the HF bulb areas (C-D). **E-G**. Hair bulb portions were scraped off for DP cell isolation. Red and green fluorescence could be seen in the collected tissues (F-G). **H-J**. No hair bulb portions could be seen on the dermal side of the collected skin biopsy after scraping. Red and green fluorescence could no longer be observed in the remaining HF portions from the dermal side of the skin (I-J). Scale bar: 1 mm for B, E, H; 200 mm for C-D, F-G, I-J.

We developed a protocol to isolate CD133 + and CD133− DP cells from full anagen HFs (Figs. 2A and S3). The back skin was collected from the mice eight days after the hair was plucked at P57 (PP8, Fig. S3A-B). Subcutaneous tissues were removed, and the skins were then floated in a solution of collagenase I, collagenase IV, and hyaluronidase with the dermal side facing down (Fig. S3C). As shown in Fig. 2B–D and S3D, HFs could be observed on the dermal side with green and red cells in the DP region in each HF. After incubating the skin for 30 minutes, the HF bulb portions could be easily scraped off using a razor blade under a dissecting microscope (Fig. 2E). As shown in Fig. 2F–G, red and green cell clusters can be seen in the hair bulb mixture under a fluorescence microscope. Afterward, no black hair bulbs remained on the dermal side (Fig. 2H and S3E-F). Red and green cells could no longer be observed in the remaining HF structures on the dermal side under a fluorescence microscope (Fig. 2l–J), revealing the removal of the hair bulb structures. The hair bulb mixture was then resuspended in collagenase solution and digested for one hour before being filtered through a 70-μm cell strainer and a 40-mm cell strainer to obtain a single-cell suspension for fluorescence-activated cell sorting (FACS) (Fig. S4A-F). We stained sorted cells using APC-labeled anti-CD133 antibody for flow cytometry analysis. As shown in Fig. S5A-C, green+; red + DP cells were indeed CD133+. Furthermore, tdTomato− DP cells (in Fig. S6B) were CD133− as shown in Fig. S6D. We also assessed the expression of CD133 in sorted green+; red + and green+; red− DP cells by qPCR. As shown in Fig. S5D, no CD133 expression was detected in green+; red− DP cells while green+; red + DP cells expressed a high level of CD133. Sorted green+; red+ (versican+; CD133+) DP fibroblasts and green+ (versican+; CD133−) DP fibroblasts were initially cultured *in vitro* for recovery and expansion.

### CD133− and CD133 + DP cells behave differently in monolayer cell culture

**Figure 3:**
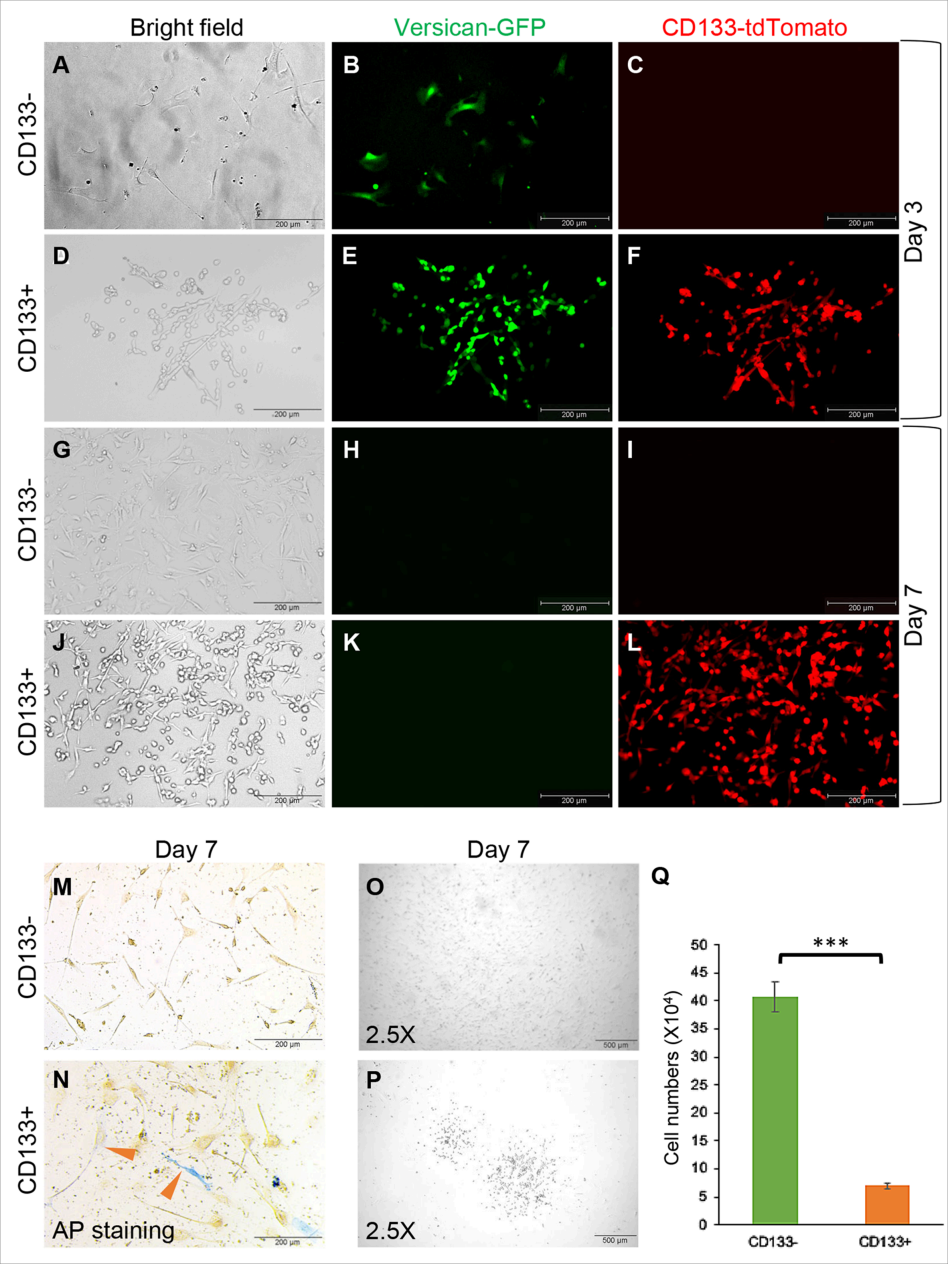
CD133+ and CD133− DP cells behave differently in monolayer cell culture. **A-F**. Representative images of monolayer cultures of isolated CD133− (A-C) and CD133+ (D-F) DP cells on day 3. (A, D) Bright-field images; (B, E) green fluorescence images; (C, F) red fluorescence images. **G-L**. Representative images showing isolated CD133− (G-I) and CD133+ (J-L) DP cells in monolayer culture on day 7. (G, J) Bright-field images; (H, K) green fluorescence images; (I, L) red fluorescence images. **M-N**. AP staining showing AP expression in CD133− and CD133+ DP cells on day 7. **O-P**. Representative images showing that CD133+ DP cells tended to grow into small colonies in monolayer culture, while CD133− DP cells spread more evenly. **Q**. Comparison of cell numbers between cultured CD133− and CD133+ DP cells on day 7. n=3. The data are presented as the mean ± SD. *P ≤0.05; **P ≤ 0.01; ***P ≤0.001; ns, not significant. Each experiment was repeated a minimum of three times. Scale bar: 200 mm for A-N; 500 μm for O-P.

Sorted mouse CD133 + and CD133− DP cells were expanded in monolayer culture in collagen-coated plates. As shown in Fig. 3B, E, green fluorescence was observed in both CD133 + and CD133− DP fibroblasts on day three, suggesting that versican, a major DP anagen protein, was actively expressed in both cell types and demonstrating the purity of the isolated DP cells. Surprisingly, green fluorescence disappeared in both CD133 + and CD133− DP cells on day seven, suggesting the loss of normal hair growth potential in both cell populations (Fig. 3H, K). As expected, red fluorescence remained in CD133 + DP fibroblasts after seven days (Fig. 3F, L). In contrast, CD133− DP cells did not express tdTomato, indicating the tight control of our inducible system (Fig. 3C, I). AP activity is a major indicator of the hair inductivity of DP fibroblasts^17^. AP staining showed that CD133 + DP cells still partially maintained AP activity after seven days of culture, while AP activity was completely lost in CD133− DP cells (Fig. 3M–N).

Morphologically, CD133 + DP cells were relatively small and compact (Fig. 3D, J), while CD133− DP cells resembled normal fibroblasts, appearing large, flat, and elongated (Fig. 3A, G). In addition, we found that CD133 + DP cells tended to grow into small colonies in monolayer culture, while CD133− DP cells spread more evenly (Fig. 3O–P). To evaluate differences in proliferation, the same number of CD133 + and CD133− DP cells were seeded in each well of a 6-well plate. After culturing for seven days, the number of CD133− DP cells was approximately six times greater than that of CD133 + DP cells (Fig. 3Q). These results suggested that CD133 + DP cells proliferated slower in monolayer culture than CD133− DP cells.

### CD133− and CD133 + DP cells behave differently in spheroid culture

**Figure 4:**
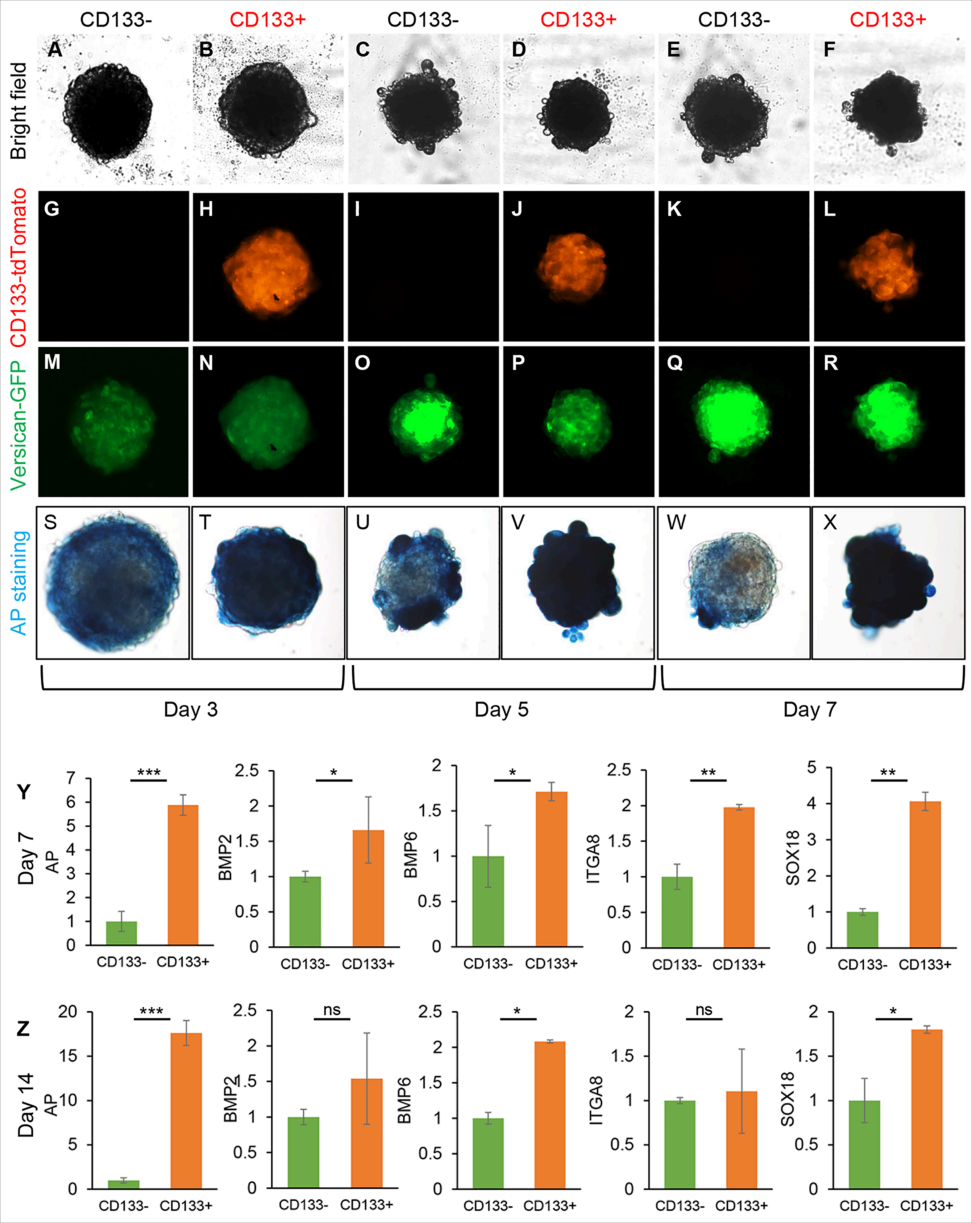
CD133− and CD133+ DP cells behave differently in spheroid culture. **A-R**. Representative images of spheroids of versican+; CD133− or versican+; CD133+ DP cells on days3, 5, and 7 obtained using a BioTek Cytation Imager 1. (A-F) Bright-field images. (G-L) Red fluorescence images. (M-R) Greenfluorescence images. **S-X**. AP staining showing AP expression in versican+; CD133− and versican+; CD133+ DP spheroids on days 3, 5, and 7. **Y-Z**. Relative DP signature gene expression levels in versican+; CD133− and versican+; CD133+ DP spheroids on day 7(Y) and day 14(Z), including *Alpl, Bmp2,Bmp6, Itga8*, *and Sox18*, by qPCR. The Y-axis represents the relative levels of indicated target gene expression with the level in versican+; CD133− DP cells set to 1. n=3. The data are presented as the mean ± SD. *P ≤0.05; **P ≤ 0.01; ***P ≤0.001; ns, not significant.

It has been shown that three-dimensional (3D) culture can maintain human DP characteristics longer compared to monolayer culture^18^; thus, we studied whether culturing CD133 + and CD133− DP fibroblasts in 3D spheroids could restore the expression of versican and AP and extend the maintenance of DP properties. Both CD133 + and CD133− DP cells aggregated to form spheroids in cell-repellent 96-well plates. As shown in Fig. 4A–F, after culturing for three days, CD133 + DP fibroblasts formed smaller and tighter spheroids than CD133− DP fibroblasts. Surprisingly, both CD133 + DP spheroids and CD133− DP spheroids started to exhibit green fluorescence on day three in 3D spheroid culture (Fig. 4M–R), suggesting that 3D culture conditions restored versican expression in DP cells. By comparison, CD133− DP spheroids exhibited stronger green fluorescence than CD133 + DP spheroids on days three and five, indicating that CD133− DP fibroblasts regain versican expression more quickly than CD133 + DP cells. TdTomato expression continued to be present in CD133 + DP fibroblasts (Fig. 4H, J, L) and never appeared in CD133− DP fibroblasts (Fig. 4G, I, K). By qPCR analysis, we confirmed that CD133− DP cells never expressed CD133 in spheroid culture and that CD133 + DP cells did not lose CD133 expression (Fig. S7).

To evaluate the hair inductivity of DP fibroblasts cultured in 3D spheroids, we analyzed AP expression. CD133 + DP spheroids displayed stronger AP staining than CD133− DP spheroids at each time point (Fig. 4S–X). To further understand whether the DP properties are maintained after spheroid culture and the differences between CD133 + and CD133− DP cells, CD133 + and CD133− DP spheroids were collected on days seven and 14 for the evaluation of the expression of DP signature genes, including *Alpl,Bmp2, Bmp6, Itga8*, and *Sox18*. As shown in Fig. 4Y–Z, CD133 + DP spheroids exhibited greater expression of *Alpl, Bmp2, Bmp6, Itga8*, and *Sox18* than CD133− DP spheroids on days seven and 14. The data further confirmed that the CD133 + DP cell population is different from the CD133− DP cell population and possesses unique DP properties that can be stimulated by spheroid culture.

### CD133 + DP cells form large dermal spheres in hydrogel

**Figure 5:**
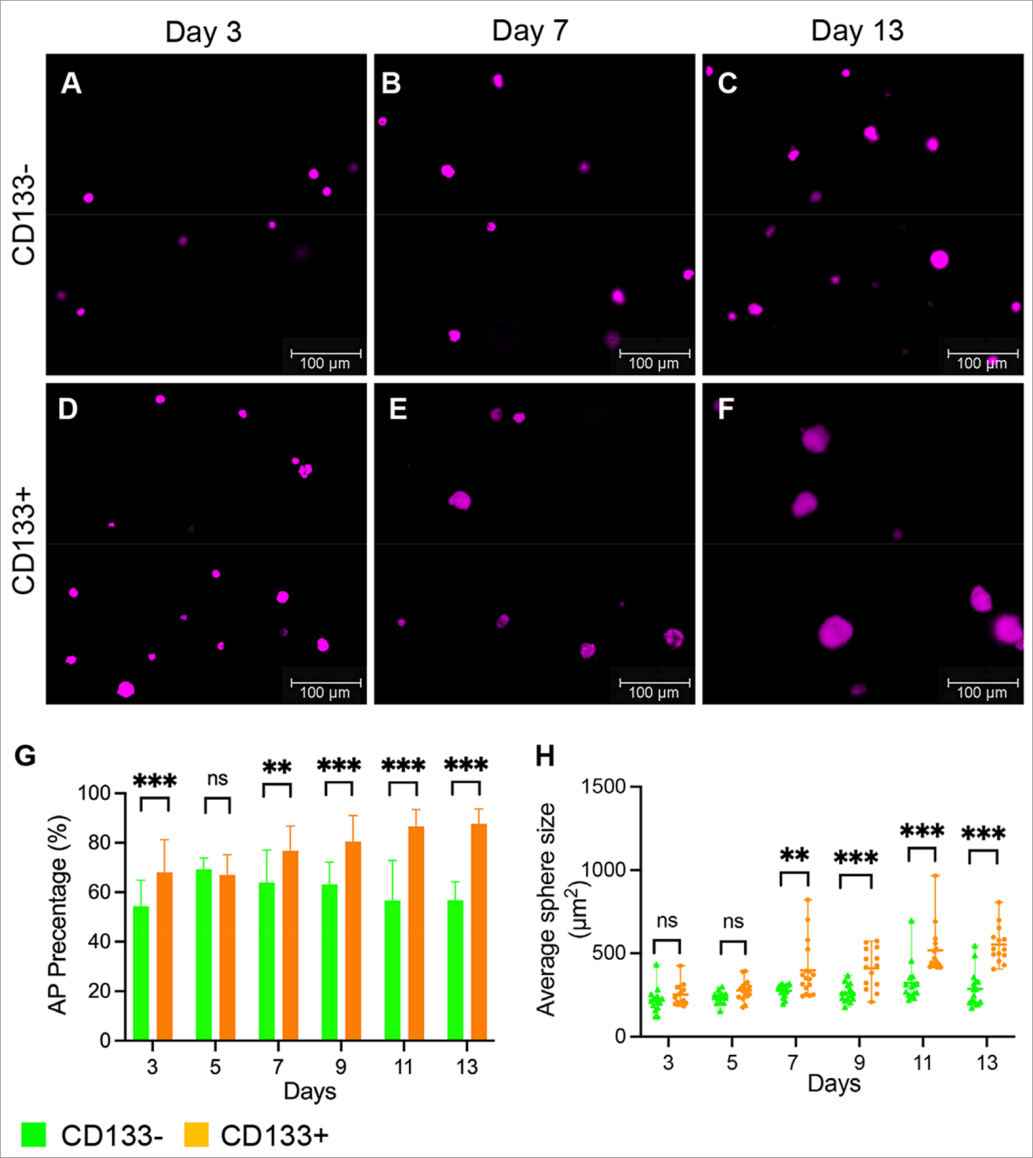
CD133+ DP cells form large dermal spheres in hydrogel. **A-F**. Representative images showing versican+; CD133− and versican+; CD133+ DP cells formed different sizes of dermal spheres in hydrogel on days 3, 7, and 13. Dermal spheres were stained purple using a VECTOR blue AP Staining Kit. Scale bar: 100 mm. **G**. The numbers of AP+ spheres and total spheres were counted using Image J to calculate the percentage of AP+ colonies among CD133− dermal spheres and CD133+ spheres. n=15. **H.** The sizes of CD133− and CD133+ dermal spheres were measured using ImageJ and compared on the indicated days. n=15. The data are presented as the mean ±SD. *P ≤ 0.05; **P ≤ 0.01; ***P ≤ 0.001; ns, not significant.

DP fibroblasts form dermal spheres in hydrogels through cell proliferation instead of aggregation^12^. Because we observed that CD133− cells proliferated more than CD133 + DP cells in monolayer culture, we further explored the proliferative capacity of CD133 + and CD133− DP cells in 3D culture by measuring how CD133 + and CD133− DP cells proliferate, form colonies, and grow in aggregates in hydrogel. CD133 + and CD133− DP cell suspensions were encapsulated in Extracel hydrogel, which consists of cross-linked gelatin and hyaluronic acid, and cultured for 13 days. As in the 3D spheroid model, we observed that red fluorescence was maintained in the CD133 + DP cell spheres, and no tdTomato expression was detected in the CD133− DP cell spheres. Both CD133 + and CD133− DP cell spheres showed green fluorescence, reflecting versican expression, as they did in spheroid culture. No significant difference in the number of spheres formed by CD133 + and CD133− DP cells was found. Furthermore, strong AP staining was detected in the CD133 + and CD133− DP spheres (Fig. 5A–F). However, the percentage of AP-positive (AP+) aggregates of CD133 + DP cells was higher than that of CD133− DP cell aggregates at any time point (Fig. 5G). When comparing the sizes of spheres formed by CD133 + and CD133− DP cells, the average size of CD133 + DP spheres became greater than that of CD133− DP spheres starting day 7 (Fig. 5H). The size of CD133− DP spheres only slightly increased during the 13-day incubation, while the sizes of CD133 + DP spheres increased significantly. The data suggest that CD133 + DP cells gain a stronger proliferative ability than CD133− DP cells when grown as 3D aggregates in a non-adherent environment.

### CD133 + DP cells, but not CD133− DP cells, drive hair follicle neogenesis

**Figure 6:**
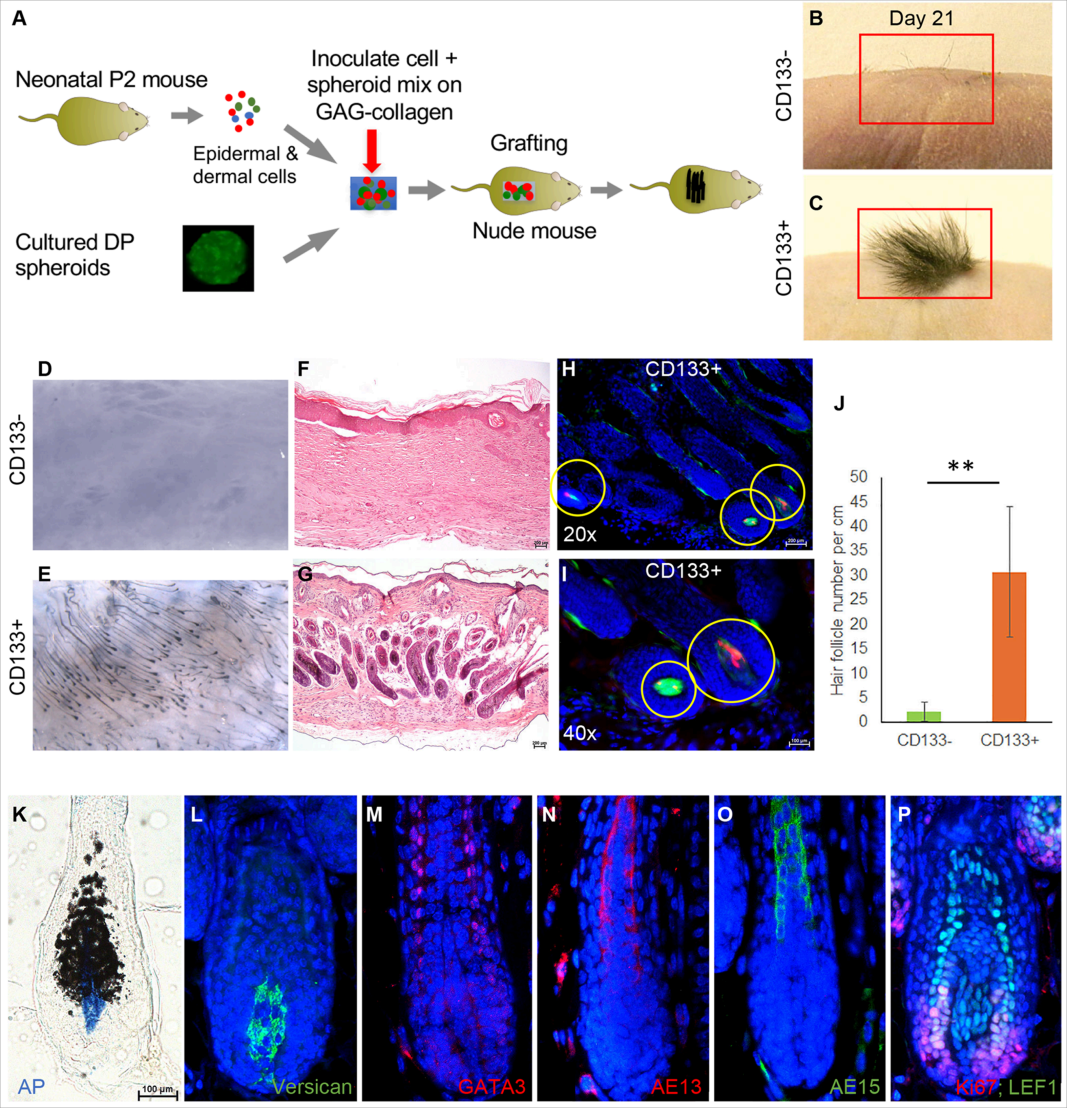
CD133+ DP cells drive hair follicle neogenesis in reconstituted skin. **A.** A schematic diagram of the skin reconstitution assay. Cultured CD133+ and CD133− DP spheroids were mixed with dermal cells and epidermal cells isolated from neonatal P2 mice, inoculated in a collagen-GAG dermal substitute, and grafted onto nude mice. **B-C.** Newly formed hairs could be observed on mice grafted with CD133+ DP cell spheroids on day 21 but not on those grafted with CD133− DP cell spheroids. n=6. **D-E.** Images of the dermal side of skin biopsies collected from the area of the healed wound on nude mice. **F-G.** H&E-stained sections of skin grafts collected from nude mice grafted with CD133− and CD133+ DP cell spheroids. **H-I.** Frozen sections of skin biopsies collected from nude mice grafted with CD133+ DP cell spheroidsshowing red and green fluorescence in the DP area of newly formed HFs (circled by yellow circles) at 20X (**H**) and 40X (**I**) magnification. **J.**Graph showing the number of HFs in skin reconstituted with CD133+ and CD133− DP spheroids. n=3. The data are presented as the mean ± SD. ***P ≤ 0.001. **K.** AP staining offrozen sections of reconstituted skin collected from nude mice grafted with CD133+ DP cell spheroids. **L-P**. Paraffin sections of reconstituted skin collected from nude mice grafted with CD133+ DP cell spheroids were processed for immunofluorescence staining using the following antibodies: versican for anagen DP, GATA3 for inner root sheath, AE13 and AE15 for hair shaft, Ki67 for hair matrix cell proliferation, and Lef1 for hair shaft progenitor cells. Scale bar: 200 mm for F-l; 100 μm for K-P.

To determine whether the *in vitro* evaluation of CD133 + and CD133− DP cells can be translated into their *in vivo* HF-driving abilities, we examined *de novo* HF formation in a skin reconstitution assay using cultured 3D spheroids (Fig. 6A). To this end, we grafted CD133 + and CD133-DP fibroblast spheroids to full-thickness wounds in athymic nude mice. Before grafting, DP spheroids were mixed with freshly isolated epidermal keratinocytes and dermal cells and inoculated in collagen–glycosaminoglycan (C-GAG) dermal scaffolds. Dressing removal day was considered day 0. New hair shafts could easily be observed in nude mice grafted with CD133 + DP spheroids on day 21 (Fig. 6C). Mice grafted with CD133− DP spheroids showed almost no newly formed hairs in the healed wounds (Fig. 6B). Black hair shafts can easily be seen from the dermal side of skin grafts derived from CD133 + DP spheroids (Fig. 6D), while only a few hair shafts can be seen in skin grafts derived from CD133− DP spheroids (Fig. 6E). H&E staining confirmed the macroscopic observation of HF formation mainly in reconstituted skin containing CD133 + DP spheroids on day 21 (Fig. 6F–G). After frozen sectioning, red− and green-labeled cells were visible in the DP region of the new HFs (Fig. 6H–I). As shown in Fig. 6J, an average of thirty HFs per cm were counted in reconstituted skin with CD133 + DP spheroids, while reconstituted skin consisting of CD133− DP spheroids had only a few HFs.

Next, we evaluated the expression of major HF structural markers, including GATA3 (inner root sheath), AE13 (hair shaft cortex keratin), AE15 (medulla), and DP markers, including AP and versican, by immunostaining newly formed HFs in healed skin wounds. As shown in Fig. 6K–L, the newly formed HFs exhibited clear AP activity and expression of versican in the DP region. The expression of GATA3, AE13, and AE15 can also be observed in their respective HF structures (Fig. 6M–O). Ki67 immunostaining was used to evaluate hair matrix cell proliferation, and LEF1 was used as a marker of HF progenitor cells. Ki67 and LEF1 double-positive cells were present in newly formed HFs in skin reconstituted with CD133 + DP spheroids (Fig. 6P). The data demonstrated that CD133 + DP cells, but not CD133− DP cells, possess initiating ability to drive HF neogenesis.

## Discussion

To improve TESS functions, cells with appendage-inducing capabilities, such as DP fibroblasts located at the base of each HF, are needed. A plethora of obstacles has plagued the use of isolated human DP cells for HF engineering. The major limitations are their extremely poor proliferative potential and inability to retain trichogenicity *in vitro*^19^. Due to the lack of ways to expand a potent mesenchymal cell population *ex vivo* that can be used to instruct keratinocytes to form HFs, bioengineering of a fully functional human TESS has not yet been achieved.

Isolated murine DP fibroblasts are able to induce HF formation even from human keratiocytes^20–23^. Consistent with previous findings^24,25^, in our study, we found that mouse DP fibroblasts were still capable of inducing HF morphogenesis after *ex vivo* expansion. These observations suggest that murine DP cells possess unique hair-inducing abilities that do not exist in human DP fibroblasts. Furthermore, DP cells require a unique three-dimensional niche to maintain trichogenicity^18^. When removed from their native habitat, murine DP cells can temporarily retain the inductive properties required to conduct trichogenesis, while human DP cells fail to do so. These functional discrepancies between human and murine DP fibroblasts indicate the importance of understanding the unique intrinsic properties of mouse DP cells in directing us to decipher the “dark matter” of the mesenchymal compartment in human HFs. One of them is the heterogeneity of the cell populations within the DP, which is underappreciated and may dictate distinct functional capabilities among DP subpopulations^26^. An example of heterogeneity within a mouse DP is reflected by CD133 expression^27^. CD133 + DP cells isolated from embryonic or adult DPs have the ability to induce new HFs *in vivo*^11,12,14^. Using an *in vitro* 3D hydrogel culture system and skin reconstitution assay, CD133 + DP cells were shown to contribute to the establishment of the DP in both primary and secondary HFs^12^. We also reported that the manipulation of canonical Wnt/b-catenin signaling in CD133 + DP cells increases hair inductivity and regulates postnatal hair growth^13,28^. Taken together, the results demonstrated that CD133 + DP cells are a unique DP cell subpopulation important for HF formation.

Nevertheless, although expressed in all mouse DPs, not all DP cells express CD133 as shown in our tracing experiment. Notably, CD133− cells used in previously published studies were presumably non-DP cells^11,12,29^, which cannot be used to address intra-DP heterogeneity based on CD133 expression. Theoretically, CD133− DP cells cannot be overlooked because they constitute a large part of the DP population. To date, due to the lack of genetic tools that can be used to isolate CD133− DP cells, the biological differences between CD133 + and CD133− DP cell populations in terms of DP characteristics and HF neogenesis have remained unclear, hindering efforts to uncover the key driving force in the DP that orchestrates their reciprocal interactions with keratinocytes to generate HFs.

Multiple approaches were adopted to validate the correlation between tdTomato and CD133 expression. The combination of versican and CD133 labeling demonstrated that the functional heterogeneity of DP cells in monolayer culture, 3D culture, and *in vivo* HF induction assays correlated with CD133 + expression. That is not to say CD133 + and CD133− DP cells are different from each other. Both CD133 + and CD133− DP fibroblasts express versican initially because they were isolated from the same anagen HFs. However, they quickly lost their versican expression in monolayer culture, which was restored when the cells were cultured in 3D spheroids, suggesting that a 3D microenvironment is needed for the maintenance of DP cell hair inductivity. Accordingly, CD133 + and CD133− cells surprisingly formed large AP-positive spheres in spheroid and hydrogel cultures, although CD133 + DP cells exhibited greater AP expression than CD133− DP cells. The results confirmed the DP identity of CD133 + and CD133− cells, despite their roles in the DP might differ.

The role of CD133 + DP cells is intriguing. A notable characteristic of CD133 + DP cells compared to CD133− DP cells is that they tend to form clusters in monolayer cultures and possess higher proliferative rates in 3D and hydrogel cultures. Our analysis also confirmed that CD133 + DP cells are superior in maintaining DP characteristics and generating HFs in skin reconstitution assay, while CD133− DP cells failed to match their capabilities. We showed that CD133 + cells greatly benefit from 3D spheroid culture to maintain a high HF-generating capacity *in vitro*. Because CD133 expression in the DP is observed prior to versican expression and is restricted to a small DP population compared to a much wider versican-expressing population, the observed phenotypes support the possibility that CD133 + DP cells are inductive cells in the DP, while CD133− DP cells are responsive, supporting DP cells. CD133 + DP cells may play a central role during the hair growth stage by activating responsive DP cells and stimulating keratinocytes. Clearly, the mechanisms underlying the biological differences between CD133 + and CD133− DP cells are still incompletely understood and need to be further investigated.

Whether CD133 itself is more than just a useful marker for a highly HF-inducing mesenchymal cell population is unclear. The lack of an observed impact of CD133 loss on HFs may indicate that CD133 is not an important regulator by itself. This is further highlighted by the absence of data supporting the expression of CD133 and other murine DP markers, such as *Sox2*, in human DP^30,31^. This discrepancy led to the speculation that CD133 expression in HFs is specific to mice^32^, however, Gay *et al*. provided supporting evidence for the transient expression of CD133 in human HF development, although its expression was confined to the placode and absent from the dermal condensate^33^.

That is not to say that CD133 expression or CD133 + DP cells are not useful at all for the purpose of engineering human HFs. What is missing in human DP cells after leaving their natural niche remains unknown. The results shown in this study suggest that murine CD133 + DP cells possess unique “trichogenic power” *ex vivo*, which may help us understand what is lost in human DP cells *ex vivo* and what is needed to be restored in human DP cells to maintain their trichogenicity. Deciphering murine CD133 + DP fibroblasts using multiomics or other genetic tools may provide us with much-needed information to improve the use of human DP cells in TESS. Taken together, studying murine CD133 + DP fibroblasts may present an opportunity that we should not overlook to uncover the secrets in HF biology.

## Materials and Methods

### Mice

*CD133-CreER*^*T2*^ (*Prom1*^*C-L*^) mice were kindly provided by Dr. Richard J. Gilbertson^34^. Versican-EGFP mice were purchased from The Mutant Mouse Resource & Research Centers (MMRRC) (Stock Number:010543-UCD)^35^. *CAG-tdTomato* transgenic mice (stock number: 007909) were purchased from the Jackson Laboratory (Bar Harbor, ME)^36^. Versican-EGFP mice, *CD133-CreER*^*T2*^ mice, and *CAG-tdTomato* mice were crossed to generate versican-EGFP; *CD133-CreER*^*T2*^; *CAG-tdTomato* mice. Mice were genotyped by polymerase chain reaction (PCR) analysis of genomic DNA extracted from tail biopsies. The presence of the *CreER*^*T2*^ transgene in the *CD133* locus was confirmed by genotyping using the forward primer, CAGGCTGTTAGCTTGGGTTC, and reverse primer 1, AGGCAAATTTTGGTGTACGG. The CD133 wild-type allele was genotyped using the above forward primer with reverse primer 2, TAGCGTGGTCATGAAGCAAC. The presence of versican-EGFP was confirmed by PCR using the EGFP forward primer, CCTACGGCGTGCAGTGCTTCAGC, and the EGFP reverse primer, CGGCGAGCTGCACGCTGCGTCCTC. The presence of the tdTomato transgene was confirmed by PCR using the forward primer, CTGTTCCTGTACGGCATGG, and the reverse primer, GGCATTAAAGCAGCGTATCC. The PCR protocol included 94°C for 3 minutes; 35 cycles of 94°C for 30 seconds, 62°C for 30 seconds, and 72°C for 40 seconds; and a final extension at 72°C for 10 minutes. All mice were housed in the Laboratory Animal Services Facility of the University of Cincinnati under an artificial 12/12 light-dark cycle and were allowed free access to standard mouse chow and water. All experimental procedures involving mice were approved by the Institutional Animal Care and Use Committee of the University of Cincinnati approved under protocol 22–08-19–01 and were in accordance with the ARRIVE guidelines. All procedures involving biosafety issues were carried out under the protocol of the University of Cincinnati Institutional Biosafety Committee 16–08-17–01.

### Transgene induction

To induce Cre recombinase activity and tdTomato expression, tamoxifen (TAM) (Sigma–Aldrich, St. Louis, MO, Cat# 85256) in corn oil (10 mg/ml) was administered to adult *versican-EGFP; CD133-CreER*^*T2*^; *CAG-tdTomato* mice by intraperitoneal (IP) injection at 1 mg/10 g body weight for seven consecutive days. For the natural hair cycle, TAM administration starts at P19. For pluck-induced hair cycling, mice were first administered TAM at P55, and dorsal hairs were removed at P57. At each indicated time point, mice were euthanized by CO_2_ inhalation and cervical dislocation for skin tissue collection. The collected skins were processed for either DP cell sorting or frozen sectioning. For lineage tracing, 5-μm-thick frozen skin tissue sections were cut and imaged using a Nikon Eclipse 80i fluorescence microscope (Tokyo, Japan). A minimum of three mice were examined for each time point.

### DP fibroblast isolation

After dorsal skin from the hair-plucking area on the mouse was collected, subcutaneous tissues were removed using a blade. The skin was disinfected with iodine, washed with phosphate buffered saline (PBS) three times, and then floated in a digestion solution consisting of 2.5 mg/ml collagenase I (Thermo Fisher, Cat# 17100–017), 2.5 mg/ml collagenase IV (Thermo Fisher, Cat# 17104–019), 0.5 mg/ml hyaluronidase (StemCell Technologies, Cat# 07461) and 1x antibiotic-antimycotic (Corning, Cat# 30–004-CL) in PBS at 37°C for 30 minutes. Afterward, the skin was placed in a 10 cm tissue culture dish with the dermal side facing up, and the tissues around the hair bulb area were scraped off using a razor blade and collected into a 15 mL conical tube. To generate a single-cell mixture, the above digestion solution was added to the hair bulb collection tube, and the tube was placed on a shaker at 37°C and shaken for one hour with manual pipetting every 10 minutes. After one hour, the reaction was stopped by the addition of cell culture medium. Cell debris and tissue aggregates were removed by filtering the cell mixture through a 70-μm cell strainer and a 40-μm cell strainer. A filtered single-cell suspension was then used for FACS to isolate versican+; CD133 + DP fibroblasts and versican+; and CD133− DP fibroblasts based on red and green fluorescence.

To confirm that red fluorescent DP cells express CD133 while green fluorescent only cells do not express CD133, filtered cells were resuspended in 100 μL of cold PBS supplemented with 1% fetal bovine serum (FBS) and incubated with anti-CD133 antibody (clone 13A4, eBioscience, Cat# 14–1331-82) at a 1:50 dilution for 30 minutes at room temperature with gentle mixing every 10 minutes. After the incubation, cells were washed with PBS to remove unbound antibody and then subsequently incubated with Goat anti-Rat IgG (H + L) secondary antibody conjugated to eFluor^™^ 660 (APC, 1:100, eBioscience, Cat# 50–4017-82) for 30 minutes on ice. Following the secondary antibody staining, cells were washed and resuspended in PBS for flow cytometry analysis. *CD133-CreER*^*T2*^+ (Green−; red−) DP cells were isolated as negative control. *Versican-EGFP+; CD133-CreER*^*T2*^ + (Green+; red−) and *CD133*-*CreER*^*T2*^*+; CAG-tdTomato+* (Green−; red+) DP cells were used as single-color controls. The cells were stained only using APC-conjugated goat anti-Rat IgG (H + L) secondary antibody were used as APC negative control.

### DP fibroblast culture

Five thousand sorted mouse DP fibroblasts were seeded in a well of a CELLSTAR 6-well plate (Greiner Bio-One, Cat# 657160) that was precoated with 1 mL of collagen coating solution (Sigma, Cat# 125 – 50) and maintained in AmnioMAX^™^ C-100 medium (Thermo Fisher, Waltham, MA) supplemented with 10% AmnioMAX ^TM C-100 supplement and 1% antibiotic-antimycotic (Corning, Manassas, VA, Cat# 30–004-CL) in a humidified incubator at 37°C with 5% CO_2_. The culture medium was replaced every other day until confluence, at which point the cells were trypsinized for expansion. At each indicated time point, the cells were imaged using a Leica DMi8 fluorescence microscope at 2.5X and 10X magnification. To count the cell number, cultured DP fibroblasts were trypsinized, collected by centrifugation at 400 g for five minutes, and resuspended in culture medium. The cells were counted manually using a hemocytometer. For AP staining, a VECTOR Blue Alkaline Phosphatase Substrate kit (Vector Laboratories, Burlingame, CA) was used according to the manufacturer`s instructions. Images of the cells were taken using a Leica DMi8 microscope at 10X magnification (Wetzlar, Germany). Cell culture reagents were purchased from Thermo Fisher Scientific unless otherwise stated.

### DP fibroblast spheroid culture

To culture DP fibroblasts in 3D spheroids, 2000 cultured mouse DP fibroblasts were trypsinized, resuspended in 50 μl of AmnioMAX^™^ C-100 culture medium, and then seeded in a well of a CELLSTAR 96-well U-bottom plate (Greiner Bio-One, Cat# 650970), which allowed the cells to aggregate and form a spheroid. The culture was incubated at 37°C in a humidified incubator with 5% CO_2_. At each indicated time point, the spheroids in each well were scanned and photographed using a Cytation 1 cell imaging multimode reader (BioTek Instruments, Winooski, VT) to track the expression of red tdTomato and EGFP. To evaluate AP expression in DP fibroblasts, the spheroids were collected on days 3, 5, and 7, and stained using the VECTOR Blue Alkaline Phosphatase Substrate kit. Images of the stained spheroids were taken using a Leica DMi8 microscope.

### Hydrogel culture

To culture DP fibroblasts in hydrogel, 1 × 10^4^ cultured mouse DP fibroblasts were trypsinized, mixed with 100μ l of Extracel hydrogel (Advanced BioMatrix, Carlsbad, CA) according to the manufacturer`s instructions, and seeded in a well of a TPP 96-well plate with flat bottom (TPP Techno Plastic Products AG, Trasadingen, Switzerland). The culture was incubated at 37°C in a humidified incubator with 5% CO_2_ until the indicated time points. The VECTOR blue AP Substrate kit was used to evaluate AP activity in DP fibroblast spheres in the hydrogels. Brightfield and corresponding florescent pictures of DP fibroblast spheres in the hydrogels were taken from the same fields using a Leica DMi8 microscope at 10X magnification. To count the numbers of CD133 + and CD133− spheres and measure their sizes at each time point, at least three random, representative pictures from each well were selected and counted using ImageJ^37^. A total of 15 pictures from three independent experiments were counted at each time point. The number and average size of spheres were counted and measured in brightfield pictures using ImageJ and analyzed using GraphPad Prism 8. The AP + sphere numbers were counted in corresponding florescent pictures. The percentage of the AP + spheres under at each time point was calculated by dividing the number of AP+ spheres with the number of spheres counted in brightfield pictures. The data are representative of three independent experiments.

### Quantitative real-time PCR

Total RNA was extracted from 10–16 spheroids using the RNeasy Micro kit from Qiagen (Hilden, Germany). For cDNA synthesis, an equal amount of RNA from each sample was reverse transcribed using a Superscript IV kit (Invitrogen, Carlsbad, CA). Quantitative real-time PCR was conducted using Power SYBR Green Master Mix (Applied Biosystems, Foster City, CA) on a StepOnePlus^™^ Real-Time PCR system (Applied Biosystems). qPCR primer pairs for CD133 (VMPS-5071), *Alpl* (VMPS-269), *Sox18* (VMPS-6211), *Bmp2* (VMPS-672), *Bmp6* (VMPS-676), *Itga8* (Forward: GGGGCGACAAGACCAACACAGA and Reverse: GCCCTCCTGCACTTCTACAGTTGA), and *Gapdh* (VMPS-7317) were purchased from Real-Time Primers, LLC (Elkins Park, PA). A qPCR run consists of an initial step of 15 minutes at 95°C followed by 40 cycles of 15 seconds of denaturation at 95°C and then 60 seconds of annealing and extension at 60°C. The expression levels of each target gene in CD133 + and CD133− DP fibroblasts were analyzed using the comparative CT method and normalized using *Gapdh*^38^. CD133− DP fibroblast samples were used as a reference to calculate the relative expression of each target gene in CD133 + samples. The data are representative of three independent experiments.

### Skin reconstitution assay

The skin reconstitution assay was performed as we previously published^14^. Briefly, the trunk skin of two-day-old neonatal mice was dissected, and the epidermis and dermis were separated by floating the skin in 0.1% dispase at 37°C for two hours. Subsequently, the epidermis was cut into fine pieces and dissociated into a cell suspension by incubation in 0.25% trypsin/EDTA for 15 minutes. The dermis was dissociated in 0.25% collagenase IV solution for 30 minutes at 37°C with manual stirring every 15 minutes using a serological pipette. The isolated cell mixtures were filtered through a 70-μm cell strainer followed by a 40-μm cell strainer to generate a single-cell suspension.

For each recipient mouse, twenty cultured mouse DP spheroids were collected on day three and mixed with 2×10^6^ and 5×10^6^ of the freshly isolated neonatal epidermal and dermal cells, respectively. The cell-spheroid mixture was seeded onto a collagen-GAG dermal substitute, which was prepared as previously reported^39^, and incubated for 30 minutes at 37°C. After a 1.5 × 1.5 cm full-thickness wound was made on the back of eight-week-old nude mice, the collagen-GAG matrix inoculated with the cell-spheroid mixture was placed on the wound, sutured, and then covered with a sterile dressing. After 10 days, the dressing was removed to inspect wound closure. Three weeks later, reconstituted skin biopsies were collected and prepared for paraffin and frozen sectioning as we previously published^13,14,28,40^.

### Skin alkaline phosphatase staining

Collected skin grafts were fixed in 4% paraformaldehyde at room temperature for eight hours followed by immersing in 10% sucrose solution at 4°C overnight and then 30% sucrose solution at 4°C overnight. Fixed skin biopsies were embedded in Tissue-Tek optimal cutting temperature (O.C.T.) compound for frozen sectioning (Sakura Finetek, Torrance, CA). Five μm-thick sections were cut, fixed in −20°C acetone for five minutes, and stained using a VECTOR blue AP Substrate kit. The staining images were taken using a Nikon Eclipse 80i microscope.

### Histology and Immunostaining

Collected skin grafts were fixed in 10% formalin for 48 hours at 4°C and embedded in paraffin blocks. For hematoxylin and eosin (H&E) staining, five μm-thick sections were cut, deparaffinized, rehydrated, and stained using an H&E stain kit (Statlab, McKinney, TX). H&E-stained sections were imaged using a Nikon Eclipse 80i microscope. The length of the skin in each 10X image was measured using ImageJ. Hair follicles were manually counted in each image and the number was divided by the skin length in each image to calculate the number of hair follicles per centimeter. Three pairs (CD133 + donor cells and CD133− donor cells) of skin reconstitution grafts were analyzed, for a total of 23 images of CD133 + skin reconstitution grafts and 21 images of CD133− skin reconstitution grafts.

For immunostaining, paraffin sections were deparaffinized, rehydrated, and then demasked using citrate buffer (pH 6.0) using the microwave heating method. After washing with PBS, the sections were blocked using 10% bovine serum albumin (BSA) in PBS at room temperature for one hour and then incubated overnight with each primary antibody at 4°C. The next day, the slides were washed three times with PBS, incubated with species-specific secondary antibodies conjugated to either Alexa Fluor 488 or 594 (Thermo Fisher, Waltham, MA) at room temperature for one hour, washed with PBS three times, and then mounted using VECTASHIELD^®^ Antifade Mounting Media with DAPI (Vector Laboratory, Burlingame, CA). The following primary antibodies were used: anti-Ki67 (Imgenex, Littleton, C0, 1:50), anti-LEF1 (Cell Signaling, Danvers, MA, 1:100), anti-versican (Millipore, Billerica, MA, 1:200), anti-GATA3 (Santa Cruz, Dallas, Texas, 1:50), anti-AE13 (1:25), and anti-AE15 (1:25). Images were taken using a Nikon Eclipse 80i fluorescence microscope.

### Statistical analysis

All quantitative data were obtained from a minimum of three independent experiments. The data were analyzed using either Microsoft Excel or GraphPad Prism 8. All the statistical analyses were performed using Student’s t-test, and the results are expressed as the means ± standard deviation (SD). Differences between means were considered statistically significant at P < 0.05.

## Data Availability

The datasets used and/or analyzed during the current study available from the corresponding author on reasonable request.
